# Dr. H. Gross’s Comparative Materia Medica

**Published:** 1897-08

**Authors:** 


### DR. H. GROSS'S COMPARATIVE MATERIA MEDICA. Edited by Constantine Hering. Second edition. Philadelphia: Bœricke & Tafel, 1897. Price, half morocco, $6, net; by mail, $6.40.

This is a quarto volume of 520 pages. It was first issued thirty years ago, and was well known and much consulted by homœopathic physicians. The edition became exhausted, and it has disappeared from the notice of the younger generation of our school so as to be almost unknown. Through the enterprise of the firm of Bœricke & Tafel it has been reproduced, and is once more a candidate for professional favor.

For the information of those who never saw the first edition we may describe it as a quarto page with double columns, the left-hand column being given to the symptoms of one remedy and the right-hand column to the symptoms of another, that the eye may discover instantly the similarity of two remedies and the points of difference. The first two remedies compared are Aconite and Apis. The next are Aconite and Arnica, then Aconite and Belladonna, then Aconite and Bryonia, Aconite and Cantharides, Aconite and Chamomilla, Aconite and China, Aconite and Coffea, Aconite and Ignatia, Aconite and Nux-vomica, Aconite and Opium, Aconite and Phosphorus, Aconite and Pulsatilla, Aconite and Rhus-tox., and lastly, Aconite and Veratrum. In the same way we find comparisons of Alumina with various remedies, Arsenic and various remedies, and so on. The whole is preceded by an introduction by Dr. Gross; a pharmaceutical key by the indefatigable Dr. Hering, and some remarks by Dr. Hering. The whole constitutes a book that must be a help to the industrious practitioner seeking the true simillimum for his patients. The labor of this search is so great that there cannot be too many helps on our book-shelves. What we fail to find by one book we may successfully get by consulting another. Doubtful points of resemblance and difference may cause hesitation in giving a remedy, but if we can consult an authoritative set of comparisons like the book now under notice we may be able to decide more quickly and confidently between the indications of two nearly similar remedies.

Most of the greater books that enabled the old masters to make their wondrous cures have gone out of print. Yet they are still needed. As was said by Dr. J. B. Bell, in his famous work on diarrhœa, "Homœopathy is not making that kind of progress that renders a whole medical library obsolete every ten years," and so these old works are still needed. Bœricke & Tafel are keenly aware of the need, and so we find them now and then reproducing some old book. Hahnemann's *Chronic*

*Diseases* was a notable one of these, and was reviewed in THE HOMŒOPATHIC PHYSICIAN for April, 1896, page 195. Now comes Gross' *Comparative Materia Medica*, and we may expect others.

